# A Rare Presentation of Precursor B-cell Lymphoblastic Lymphoma in a Child

**DOI:** 10.7759/cureus.3238

**Published:** 2018-08-31

**Authors:** Zarmina Javed, Faiza Hanif

**Affiliations:** 1 Internal Medicine, Shifa College of Medicine/Shifa International Hospital, Islamabad, PAK; 2 Pathology, Shifa International Hospital Islamabad, Islamabad, PAK

**Keywords:** omental nodularity, precursor b -cell lymphoblastic lymphoma, children

## Abstract

Precursor B-cell lymphoblastic lymphoma, a type of non-Hodgkin lymphoma, is common in children. It is mostly extranodal; skin, bone and soft tissue are more often involved. However, isolated peritoneal presentation is rare. In this article a unique pediatric case of isolated omental precursor B-cell lymphobastic lymphoma is presented.

## Introduction

Lymphoblastic lymphoma, a subtype of non-Hodgkin lymphoma, is a neoplasm of immature or precursor lymphoid cells. In children, 30% of non-Hodgkin lymphomas are lymphoblastic lymphomas, and among these approximately 15% are precursor B-cell lymphomas [1].

Precursor T-cell lymphoblastic lymphoma usually presents with painless lymphadenopathy and mediastinal mass while precursor B cell type is commonly extranodal with skin (33%) being the most frequently involved site followed by lymph nodes (22%), bone (19%) and mediastinum (5%) [2].

Lymphoblastic lymphoma has a preference for the bone marrow, with a reported frequency at diagnosis of 21% as well as a reported frequency of 5–10% for the central nervous system. Central nervous system involvement is more commonly associated with relapse, especially in cases without adequate central nervous system prophylaxis [3]. Liver, spleen, and testes are some of the other sites that are rarely involved.

This article reports an unusual presentation of precursor B-cell lymphoblastic lymphoma in an eight-year-old girl. She presented with massive ascites and after an extensive workup ultimately on omental biopsy and histopathology the diagnosis of precursor B-cell lymphoblastic lymphoma was established.

During literature review multiple case reports on adult peritoneal lymphomatosis were found. One of them reported a case of relapsed B cell-acute lymphoblastic leukemia/lymphoma as an isolated omental mass [4].

However, the purpose of reporting this case is to identify omentum as a rare site of precursor B-cell lymphoblastic lymphoma in children.

Informed consent statement was obtained for this study.

## Case presentation

An eight-year-old girl presented with massive ascites. Two months ago she developed fatigue, abdominal distention and weight loss of 10–15 pounds over a month. The patient did not have any significant previous medical history. She was taken to a primary care hospital where abdominal tuberculosis was suspected and she was started on anti-tuberculosis medications. Despite treatment her symptoms did not improve. Hence, she was transferred to a tertiary care hospital.

On examination, her vitals were normal. Abdomen was distended without any indication of peritonitis or perforation and bowel sounds were normal. There were no palpable lymph nodes.

She was given supportive care and detailed lab workup was started. Peripheral blood count was unremarkable except for hemoglobin of 14.6 g/dL with low red cell indices (consistent with iron deficiency anemia as serum iron was low as well) and a platelet count of 641,000/micro liter. Peripheral smear showed hypochromia, anisocytosis and microcytosis, few reactive lymphocytes and increased platelets. Routine lab tests were normal except for C-reactive protein test (CRP) of 24.27 mg/L (normal is up to 5 mg/L). Liver function tests and coagulation profile were normal. Ascitic tap was positive for red blood cells with a total lymphocyte count of 122/cumm (30% polymorphs and 70% lymphocytes), protein 2.6 g/dL and lactate dehydrogenase (LDH) 156. Serum-ascites albumin gradient (SAAG) was less than 1.1 g/dL. Occasional pus cells were seen on peritoneal fluid examination but no growth was observed on culture. Ascitic fluid cytology showed mature lymphocytes and reactive mesothelial cells but no atypical cells. Chest X-ray was normal and to rule out suspected abdominal tuberculosis Mycobacterium tuberculosis DNA by PCR was done which came out to be negative.

**Figure 1:**
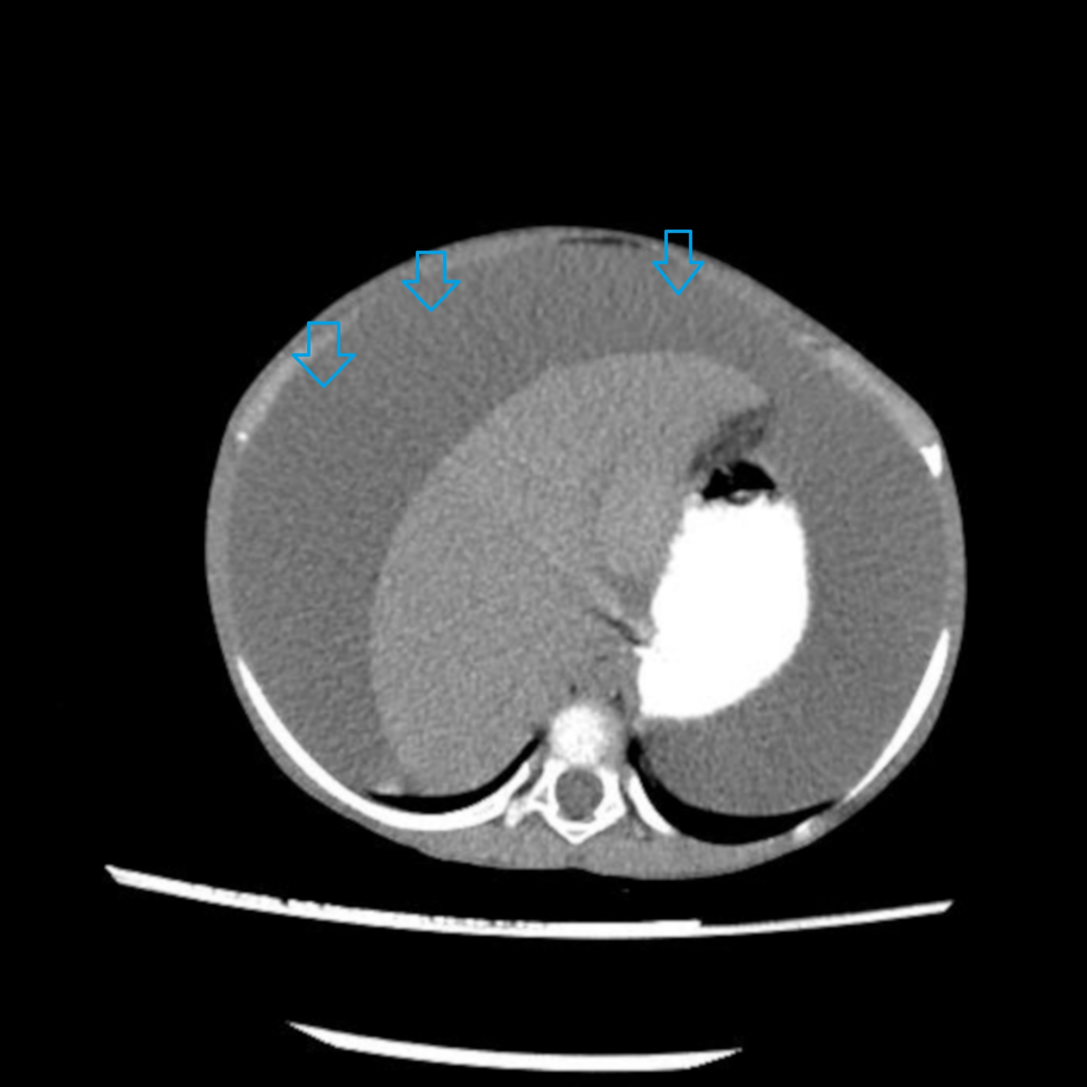
Computed tomography (CT) scan of abdomen with contrast showed gross ascites (arrows).

**Figure 2:**
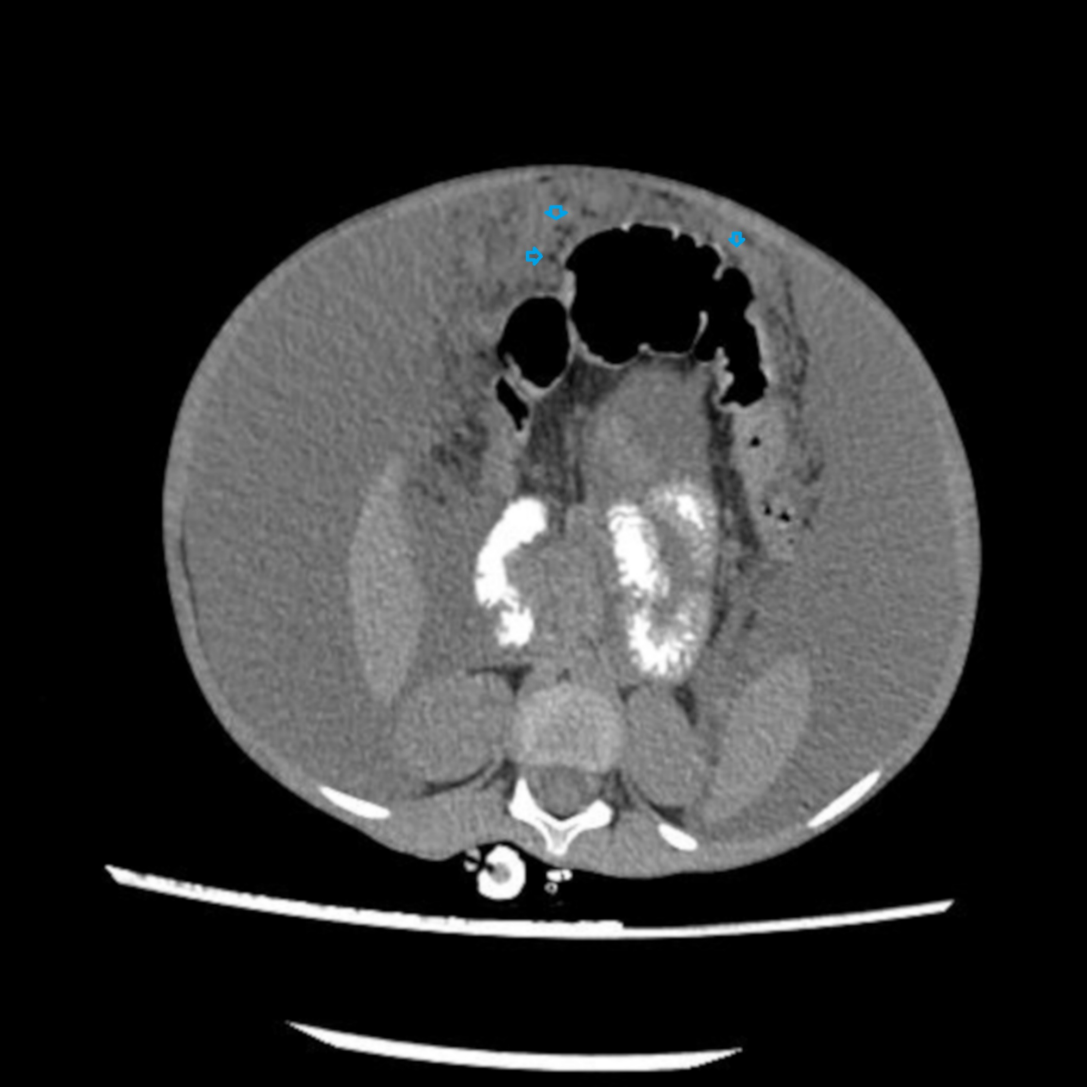
Computed tomography (CT) scan of abdomen with contrast showed omental thickening and nodularity (arrows).

**Figure 3:**
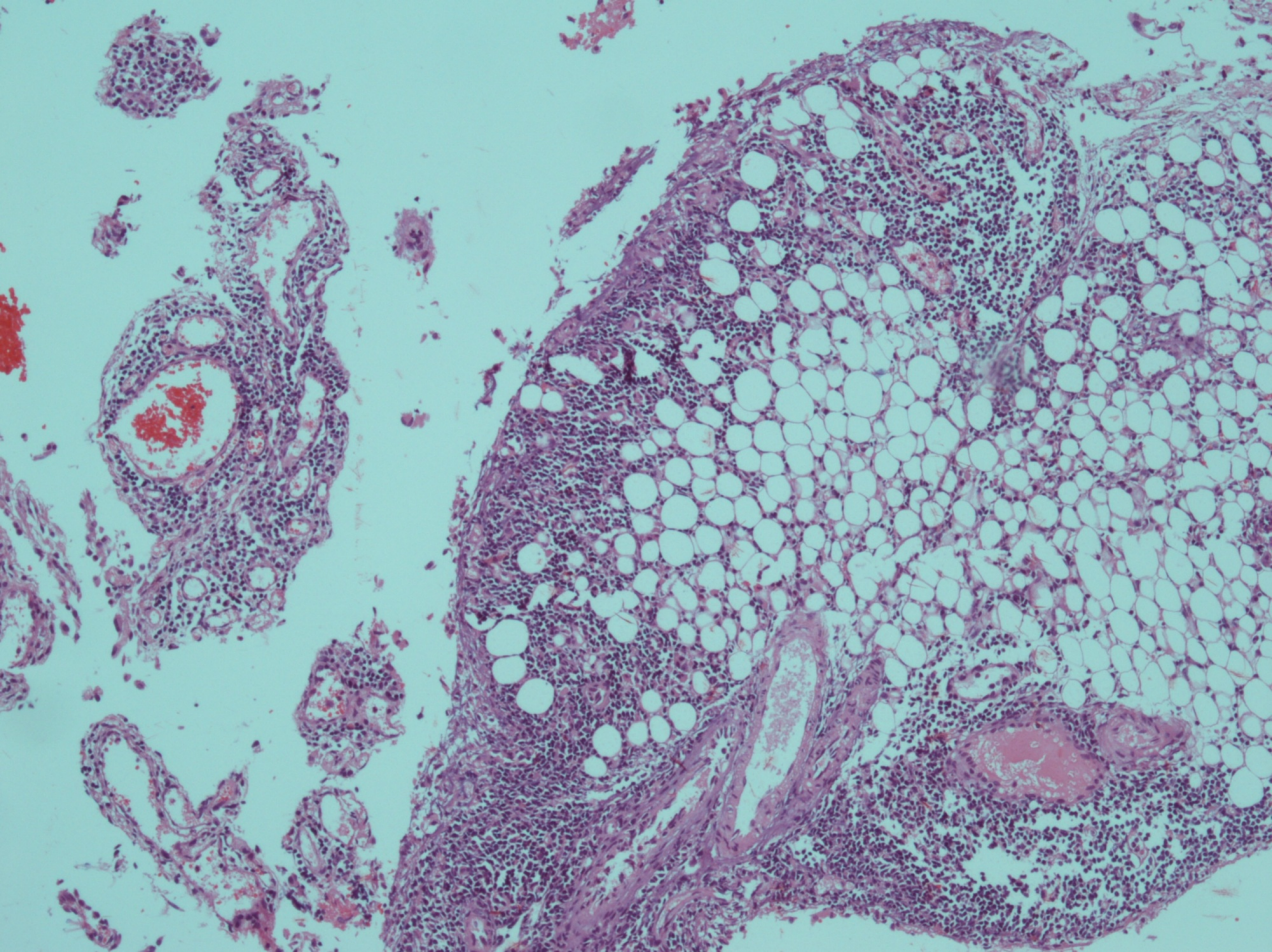
Haematoxylin and eosin (H&E) section of omental biopsy showed infiltration of fatty tissue by neoplastic blast cells (× 10).

**Figure 4:**
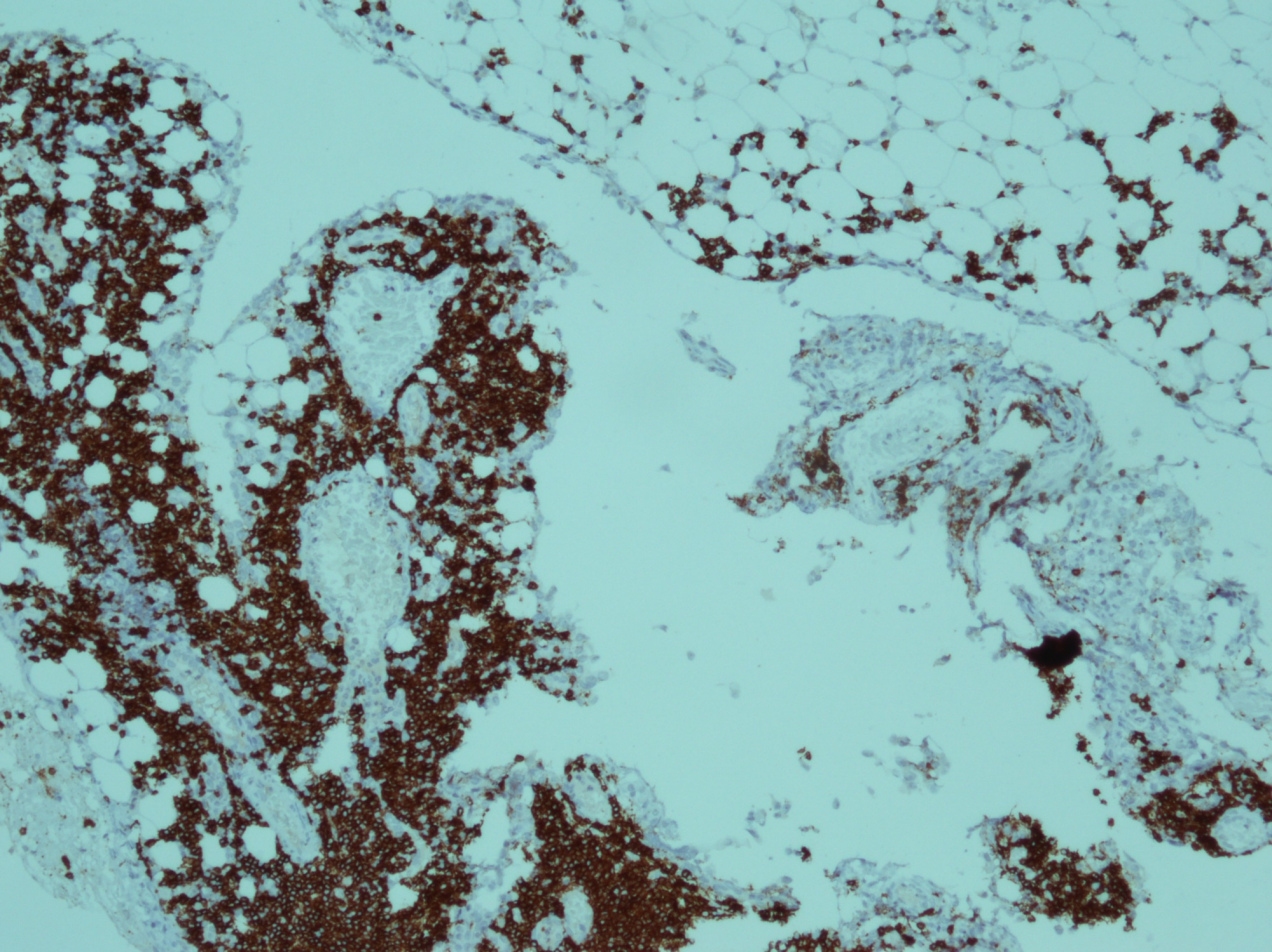
Immunohistochemical stain for CD20 showed membranous positivity in blast cells (x 40).

**Figure 5:**
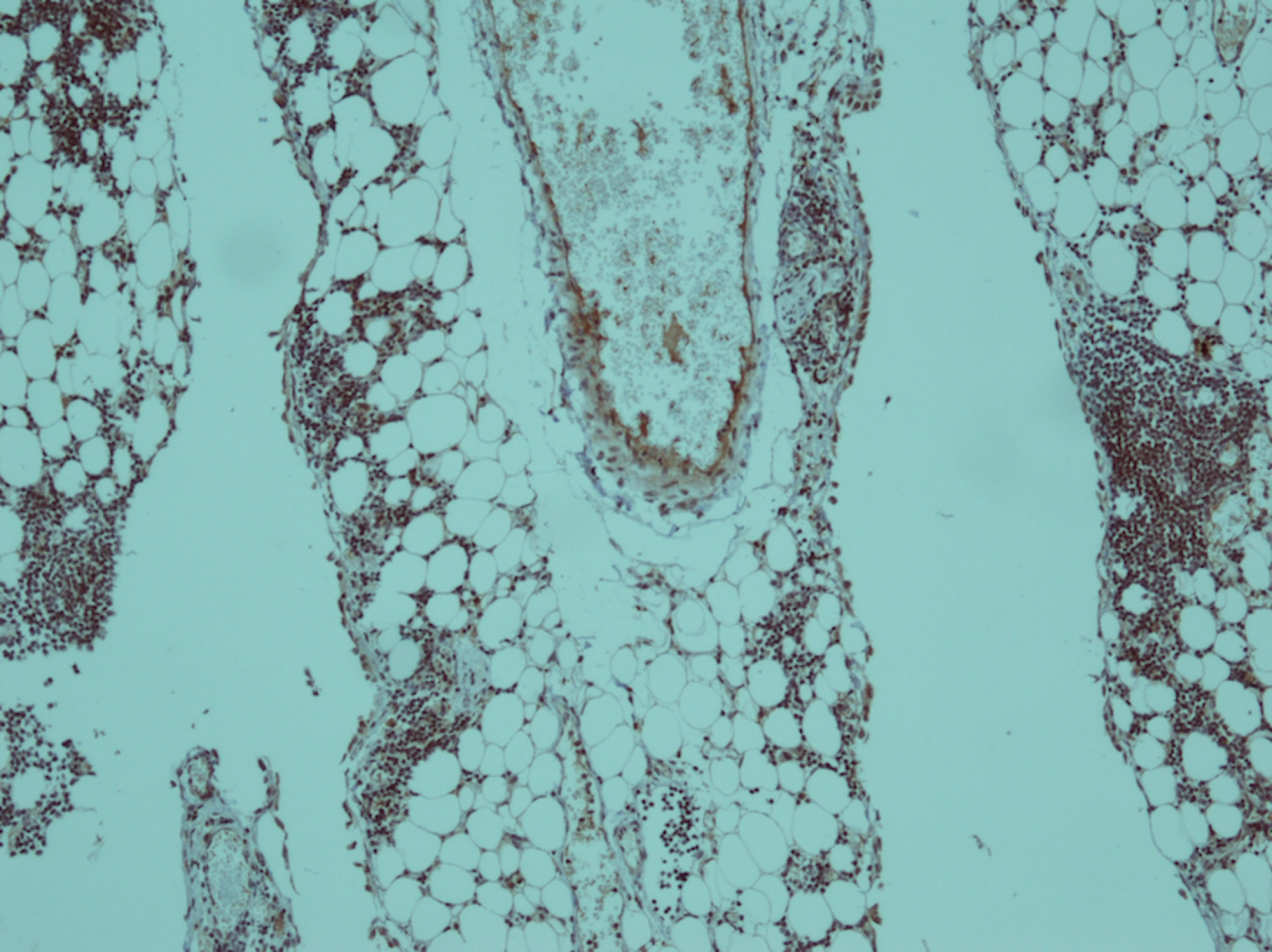
Immunohistochemical stain for Tdt showed nuclear positivity in blast cells (x 10).

Ultimately CT abdomen showed gross ascites with omental thickening and nodularity (Figures 1-2). A laproscopic omental biopsy was performed. Grossly omentum and gut loops appeared normal. However, omental biopsy showed atypical lymphoid infiltrate (Figure 3). Immunohistochemistry showed lymphocytes positive for CD20 and Tdt while CD10 negative (Figures 4-5). In correspondence with these results precursor B-cell lymphoblastic lymphoma was diagnosed. After discussing the biopsy report with the child’s parents CHOP therapy has been initiated.

## Discussion

Lymphoma is the third most common type of malignancy in children, followed by acute leukemia and central nervous system tumors [5]. Most of these are of the non-Hodgkin variety. According to the National Cancer Institute (NCI) formulation, childhood non-Hodgkin lymphomas can be classified into three types: lymphoblastic lymphomas, small noncleaved cell lymphomas (Burkitt lymphomas and non-Burkitt/Burkitt-like lymphomas) and large cell lymphomas. Amongst these lymphoblastic lymphoma is the second most frequent subtype of non-Hodgkin lymphoma (NHL) in children and adolescents [6]. Further lymphoblastic lymphomas are segregated into precursor T cell and precursor B cell lymphoma according to their lineage.

Patients with precursor T-cell lymphoblastic lymphomas usually present with lymphadenopathy and mediastinal mass. Pleural effusion may be present. Mediastinal mass can cause breathing and swallowing difficulties, and obstruction of superior vena cava. Abdominal involvement is rare while bone marrow involvement is common in precursor T-cell type.

Unlike precursor T-cell lymphoblastic lymphoma, precursor B-cell lymphoblastic lymphoma commonly presents at extranodal sites. Mediastinal and bone marrow involvement is rare [2].

Peritoneal lymphomatosis is a rare presentation of lymphoma. Usually lymphoma does not involve the omentum, a peritoneal surface; because it is a fibrofatty tissue lacking normal lymphoid elements [7]. According to literature speculation B-cell acute lymphoblastic lymphoma/leukemia cells with greater 5T4 oncofetal antigen transcript are attracted by omentum and ovaries. Bone marrows from relapsed patients have a considerably higher proportion of these 5T4 positive leukemic blasts [4].

However, the child in this case presented with isolated omental precursor B-cell lymphoblastic lymphoma for the very first time, ruling out relapse. Secondly, the very few adult cases of isolated peritoneal lymphomatosis found in literature review were mostly related to diffuse large B-cell lymphoma type of non-Hodgkin lymphoma.

## Conclusions

Isolated omental presentation of precursor B-cell lymphoblastic lymphoma is very rare but could be a possibility. Hence, patients with massive ascites of unknown origin should be considered for omental biopsy to rule out a rare presentation of precursor B-cell lymphoblastic lymphoma.
